# The Increase of Osteoporotic Hip Fractures and Associated One-Year Mortality in Poland: 2008–2015

**DOI:** 10.3390/jcm8091487

**Published:** 2019-09-18

**Authors:** Wojciech Glinkowski, Jerzy Narloch, Krzysztof Krasuski, Andrzej Śliwczyński

**Affiliations:** 1 Centre of Excellence “TeleOrto” for Telediagnostics and Treatment of Disorders and Injuries of the Locomotor System, Medical University of Warsaw, 02-005 Warsaw, Poland; w.glinkowski@gmail.com; 2 Department of Medical Informatics and Telemedicine, Medical University of Warsaw, 02-091 Warsaw, Poland; 3 Faculty of Mathematics and Information Science, Warsaw University of Technology, 00-662 Warsaw, Poland; kfk@go2.pl; 4 National Health Fund, 02-390 Warsaw, Poland; andrzej.sliwczynski@nfz.gov.pl; 5 Satellite Campus in Warsaw, University of Humanities and Economics in Lodz, 01-513 Warsaw, Poland

**Keywords:** osteoporosis, mortality, hospitalization, national, hip fracture, hip

## Abstract

Introduction: Hip fractures are a worldwide public health issue associated with significant mortality. Previous Polish studies reported an increasing trend in the number of hip fractures for both men and women, although lower than most other European countries. Materials and Methods: The Polish National Database was analyzed to examine osteoporotic hip fractures in the population aged 50 and over. Hip fracture incidence, rate, one-year mortality, and postoperative length of hospitalization were analyzed using the national health system data. Hospital discharge registry ICD-10 codes were reviewed from 2008 to 2015. Results: The incidence of hip fractures increased in both women and men by 19.4% and 14.2%, respectively. The female to male fracture ratio was 2.46. Mean postoperative hospitalization decreased from 13.6 to 11.2 days. The one-year mortality ranged between 30.45% to 32.8% for men and 26.2% to 28% for women. Of note, women aged 80–89 had the highest one-year mortality, 50.7%–55.4% after femoral neck fracture and 53%–58.5% after a pertrochanteric fracture. Conclusions: Hip fractures in Poland are increasingly more prevalent in the aging population. The unfavorable trends observed in this study indicate higher annual mortality after hip fracture, compared with other European countries.

## 1. Introduction

Hip fracture (HF) is a common condition regarded as a worldwide public health issue, leading to disability and associated with significant mortality. Hip fractures can lead to poor health outcomes and worse quality of life expressed as a substantial loss of healthy life-years in older adults [1]. The epidemiology of hip fractures is characterized by a high burden of disease, local differences, temporal trends, distinct high-risk populations, and many established risk factors. Osteoporosis impacts on the hip fracture risk. Most cases of hip fractures occur due to low-impact trauma in patients with weak bone. Hip fracture patients are at an increased risk for both mortality and subsequent osteoporotic fractures [2,3]. Hip fractures belong to a group of all types of surgical diseases that consume the most care throughout the continuum of care. Early mortality is highest after HF and remains so for several years. Studies in Poland noted the increasing trend in the total number of hip fractures as well as crude and standardized rates for both women and men [4]. The incidence of hip fractures in 2005 was [5] one of the lowest in Europe. However, HF risk in Poland [6] and four more countries (New Zealand, Romania, France, and Turkey) were considered to be low in men and intermediate for women. Hip fractures occur most frequently at 75–79 years of age for both sexes. Most frequently HFs occur in women. There are several national epidemiologic studies [7,8,9], but many reports consider regional hospitals in various countries [4,10,11].

This study aims to analyze osteoporotic fractures in the population of Poland regarding incidence, rate, length of hospitalization, and mortality risk within one year after fracture using data representative for the national health system for the population 50 years and older. 

## 2. Materials and Methods

The Polish hip fracture data was reviewed retrospectively in a study for Poland for the period between 2008 and 2015. The data were obtained from National Health Fund (NHF) and National Institute of Public Health—National Institute of Hygiene. Hospital discharge registry data of patients (men and women) over the age of 50 admitted and hospitalized in Poland contained ICD-10 (International Statistical Classification of Diseases and Related Health Problems, 10th revision) codes for proximal femur fractures (S72.1—cervical, S72.1—pertrochanteric, other S72). Data regarding outpatient treatment (including rehabilitation and long-term care) and repeated admissions were excluded from the study.

NHF is a state organizational unit performing the role of a payer in the Polish healthcare system, with the funds from compulsory health insurance contributions. The authors assumed that all patients were referred for treatment to hospitals contracting with the NHF for the treatment of fractures. The percentage of patients with HFs treated in private hospitals not reported to NHF was considered negligible, because these fractures concern a group of patients at retirement age. Also, it was supposed that the same treatment method applies in such cases regardless of the place where the treatment was provided.

The at-risk population data, as categorized by year, gender, and age (by 5-year intervals starting at 50–54 and ending at >100 years) were obtained from Statistics Poland (Statistics Poland, Basic Data) [12].

A literature search was performed in Medline, Web of Science, Scopus, and PubMed with keywords such as a proximal femur, hip fracture, epidemiology, mortality, hospitalization to obtain comparable discussion material.

Due to the national nature of the data, all trends were considered significant. Linear regression models were used to examine the trends in hip fracture incidence and mortality. A *p*-value of <0.05 was considered statistically significant. Calculations were made using Microsoft Office—Excel 2011 (Microsoft Corporation, Redmond, WA, USA). 

For this type of study informed consent or ethics committee board decision was not necessary.

## 3. Results

### 3.1. The Incidence of HFs

Between 2008 and 2015, 289,230 proximal femur fractures were recorded in men and women aged 50 years or over (205,687 in women, 83,543 in men, respectively). In this period, femoral neck fractures were slightly more prevalent than pertrochanteric fractures (112,595 cases vs. 111,209 cases). Yearly incidence rates for femoral neck and pertrochanteric fractures in men and women are collected in Table 1.

Standardization made according to the “European” population yielded rates of proximal hip fractures; these are collected in Table 2.

**Figure 1:**
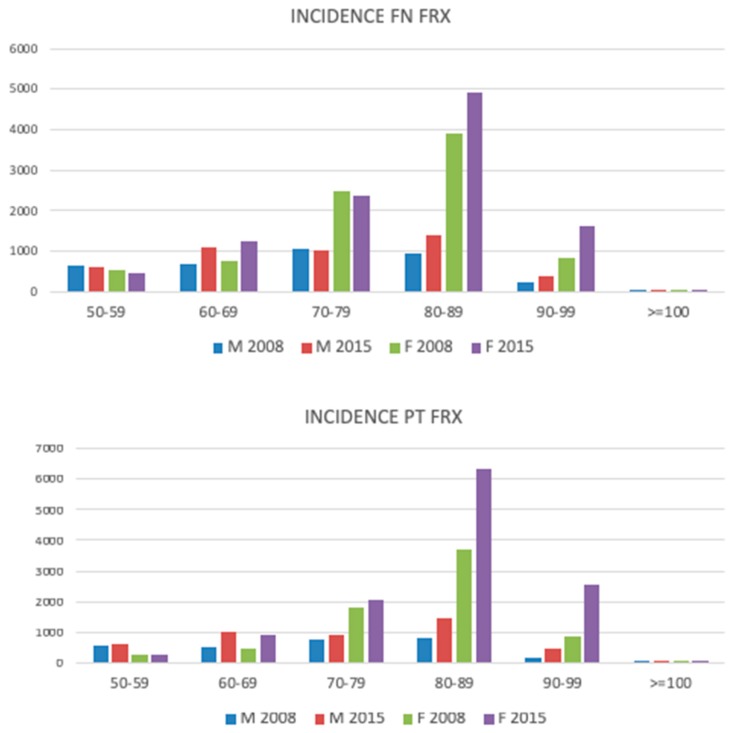
Incidence of the femoral neck (FN) and pertrochanteric (PT) fracture in different age groups, compared between men and women, in the years 2008 and 2015.

**Figure 2:**
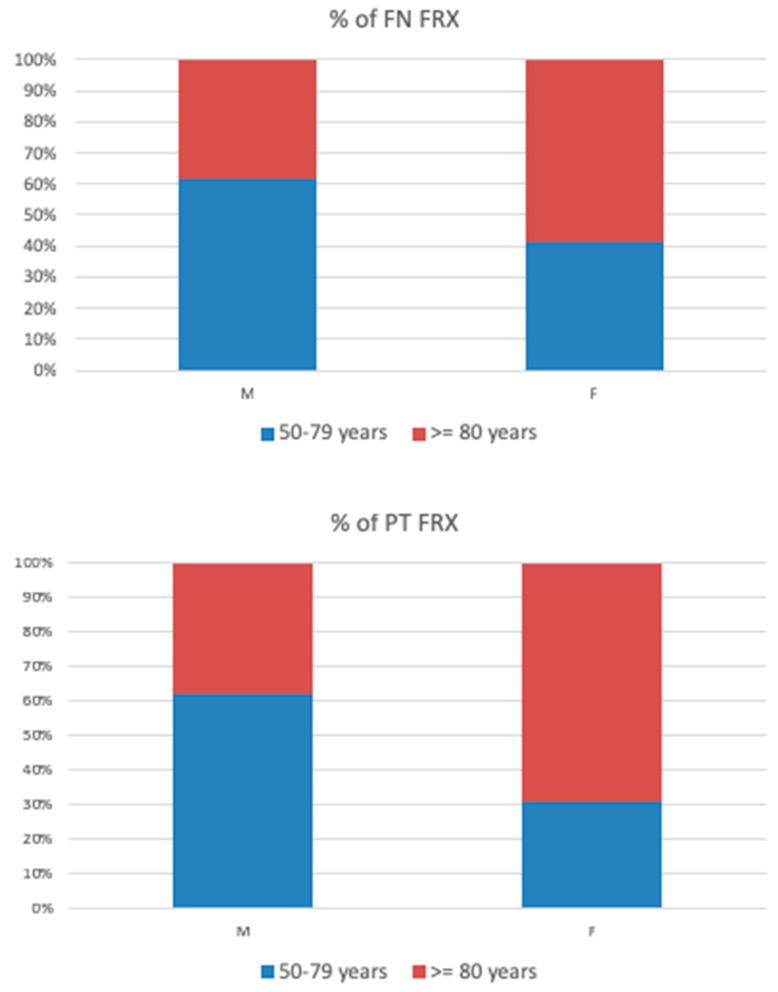
Percentage of the femoral neck (FN) and pertrochanteric (PT) fractures in age groups 50–79 years vs. ≥80 years.

The incidence of HFs increased with advancing age, with a peak at 80–89 years of age, for both men and women, and then decreased (Figure 1). A similar trend was seen in the femoral neck (ICD10—S72.0), pertrochanteric (ICD10—S72.1), and other femoral fractures (ICD10—other S72). Over one-half of all femoral neck fractures occurred in women aged 80 years or over (58.7%), while in men they accounted for 37% (Figure 2).

**Figure 3:**
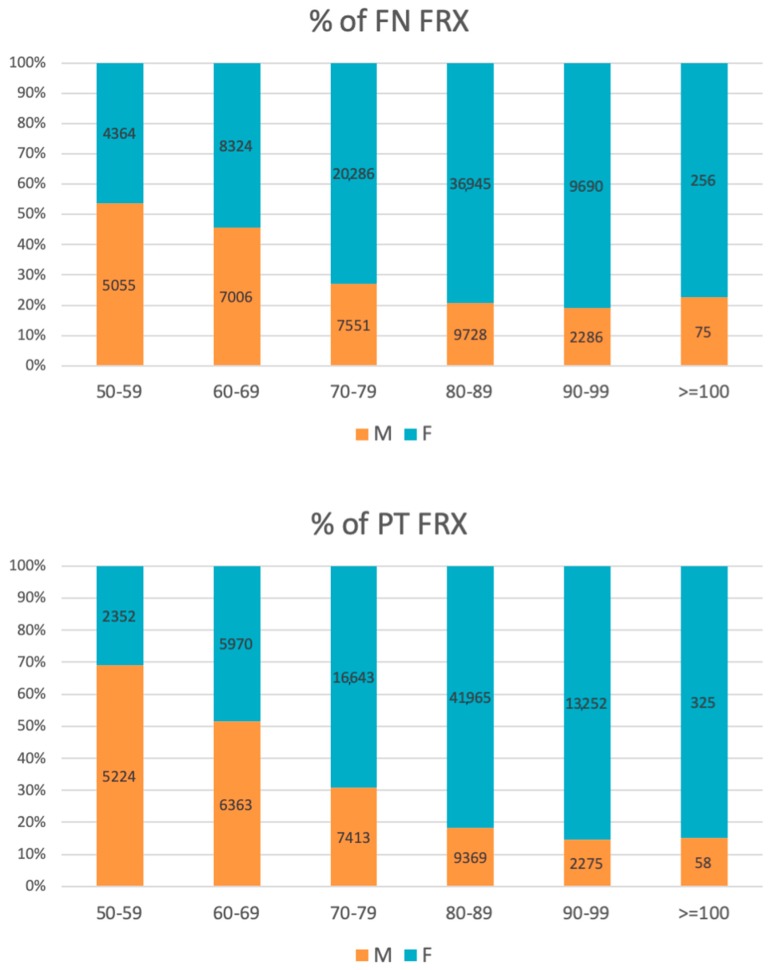
Percentage of the femoral neck (FN) and pertrochanteric (PT) fractures in all age groups (number of patients placed in bars).

In younger patients (50–59 years old) men outnumbered women in all ICD 10 categories across the study period; at the same time pertrochanteric fractures were more prevalent among men up to 69 years of age, except for the year 2010. In the following age groups, the relationship reversed in favor of women, with the absolute peak difference in a subgroup of patients aged 80–89 years (Figure 3).

The overall female to male proximal femur fracture incidence ratio was 2.46. 

**Figure 4:**
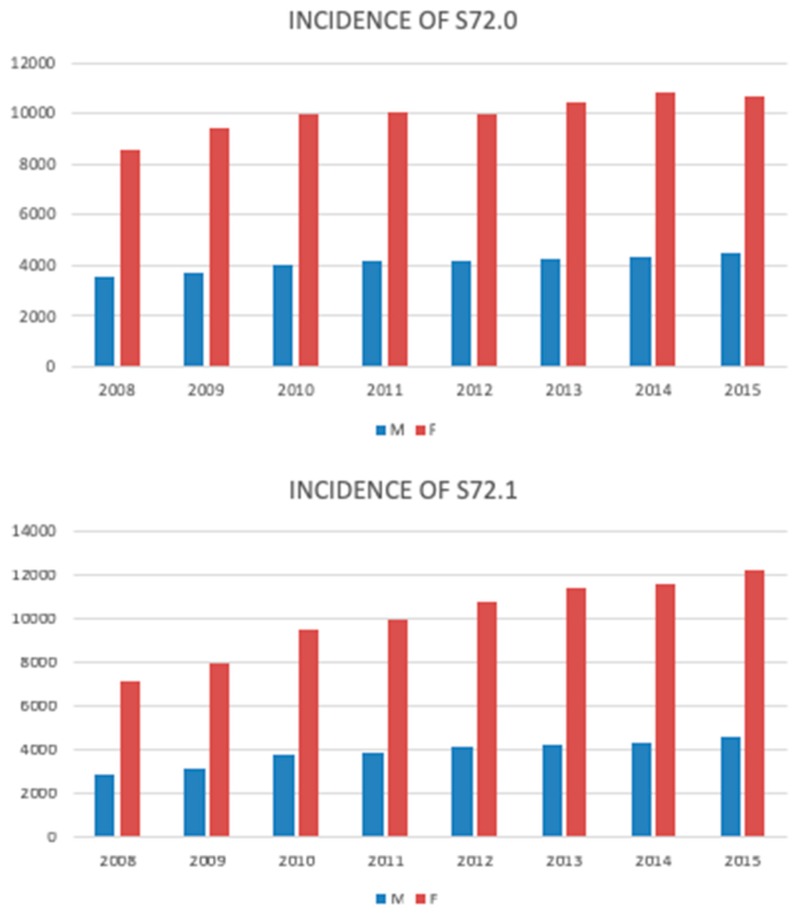
The incidence of the femoral neck (S72.0) and pertrochanteric fracture (S72.1) in men and women during the study period (2008–2015).

Over the eight years, the incidence of HFs increased each year steadily from 32,636 cases/year to 39,783 cases/year in 2008 and 2015, respectively (an increase by 18%, on average an additional 893 cases/year). Gender-specific trends revealed different growth dynamics for men and women, while both increased each year. The incidence rate increase was higher for women (19.4% vs. 14.2%). The overall trend was reflected in both the femoral neck and pertrochanteric fractures (Figure 4).

### 3.2. Hospitalization due to HFs

**Figure 5:**
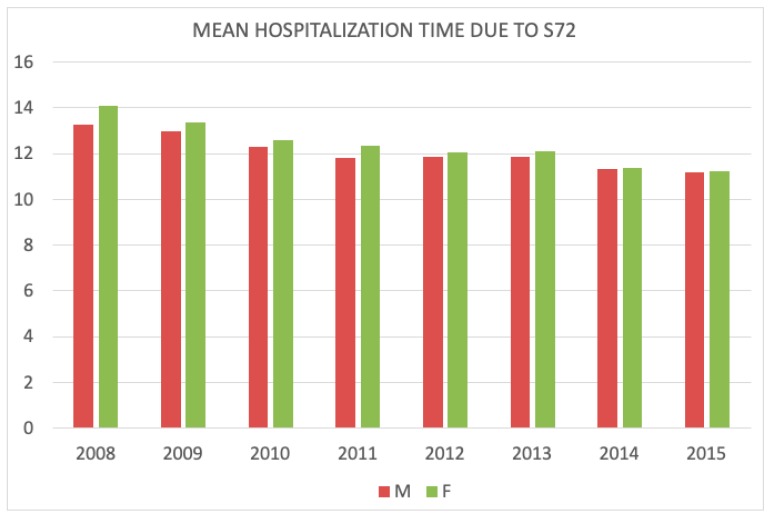
Mean hospitalization time due to HF (hip fracture) in men and women during the study period.

Mean hospitalization time decreased over the study period. In 2008 men spent, on average, 13.2 days in a hospital, while women were hospitalized for 14.1 days. Over time, the hospital stay (LOS—length of stay) has diminished. The difference between the hospitalization time of men and women has also decreased and was equalized to 11.2 days in 2015. Average hospitalization time shortened of 0.25 days/year for men, and 0.36 days/year for women (Figure 5).

**Figure 6:**
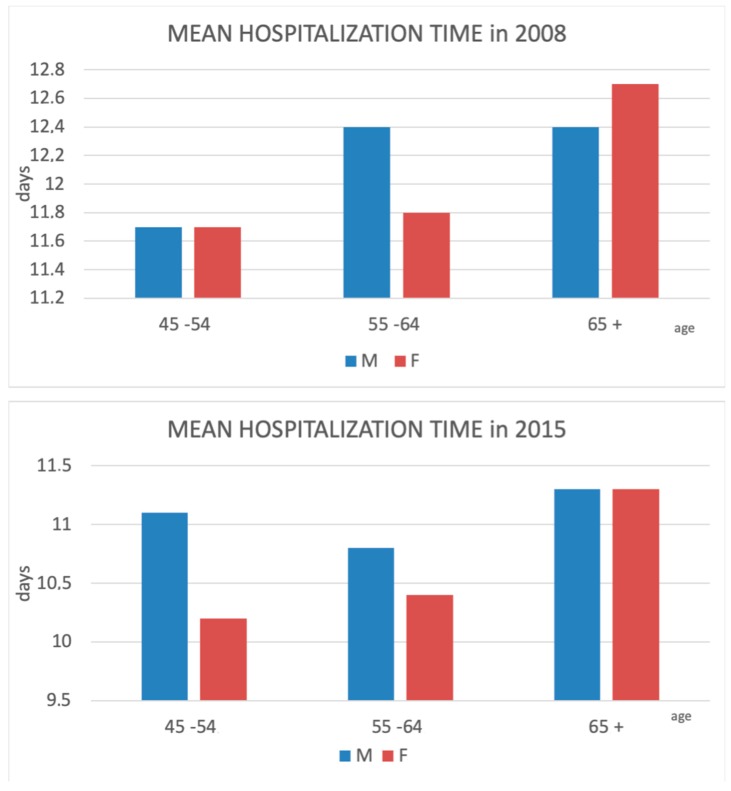
Comparison between hospitalization time due to HF (hip fracture) in different age groups at the beginning and end of the study period.

Regarding the age-group-specific observations, the length of stay increased with advancing age across the study period for both men and women (Figure 6). 

**Figure 7:**
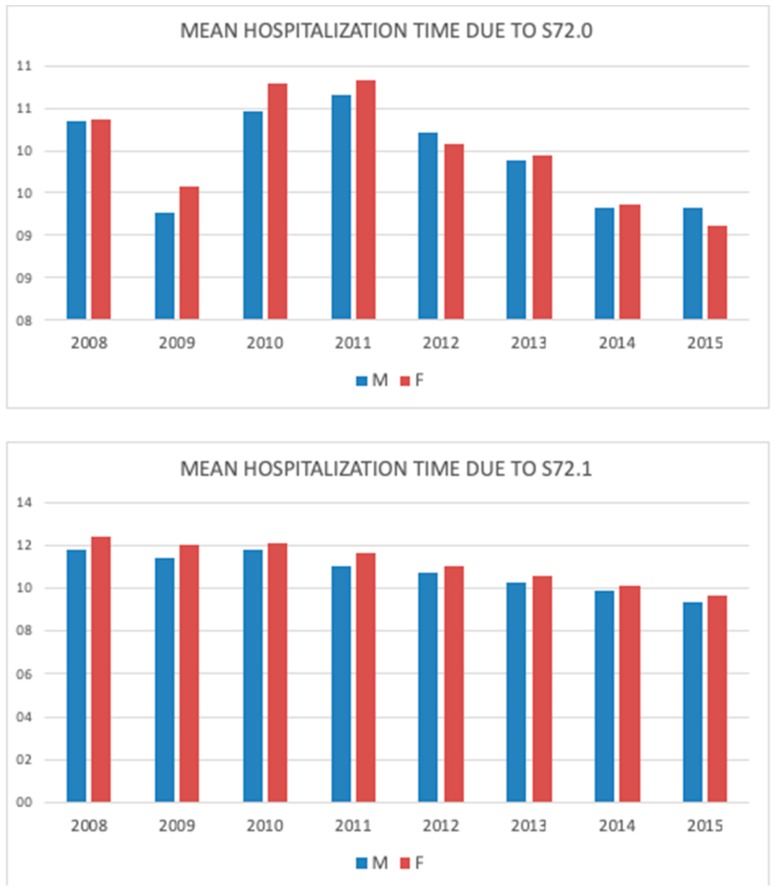
Mean hospitalization time due to the femoral neck (S72.0) and pertrochanteric fracture (S72.1) in men and women during the study period (2008–2015).

For femoral neck fractures, hospitalization time increased between 2008 and 2011, reaching its peak duration at 10.7 days for men, and 10.8 days for women. It fell subsequently to 9.3 and 9.1 days for men and women, respectively. The pertrochanteric fractures subgroup reflected the general trend of steady decrease, with 9.3 and 9.7 days for men and women, respectively (Figure 7).

**Figure 8:**
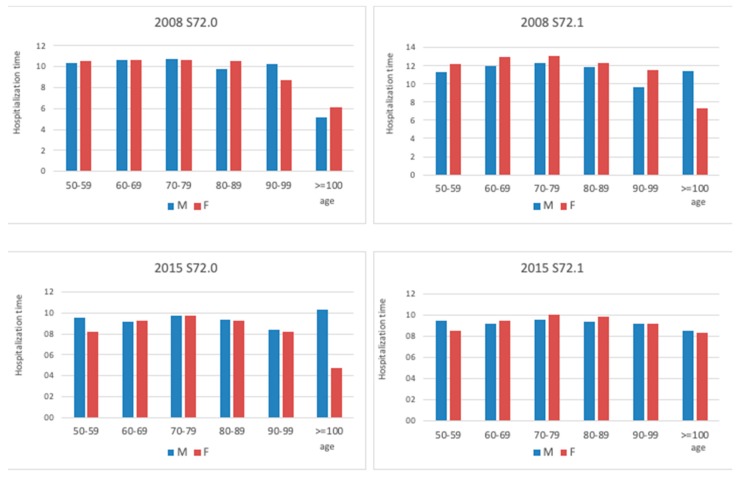
Comparison between hospitalization time due to the femoral neck (S72.0) and pertrochanteric fracture (S72.1) in different age groups at the beginning and end of the study period.

Age subgroups in men and women aged 50 years or over generally mirrored each other in trends across the study period, and also reflected the overall observations described above (Figure 8).

### 3.3. Mortality

**Figure 9:**
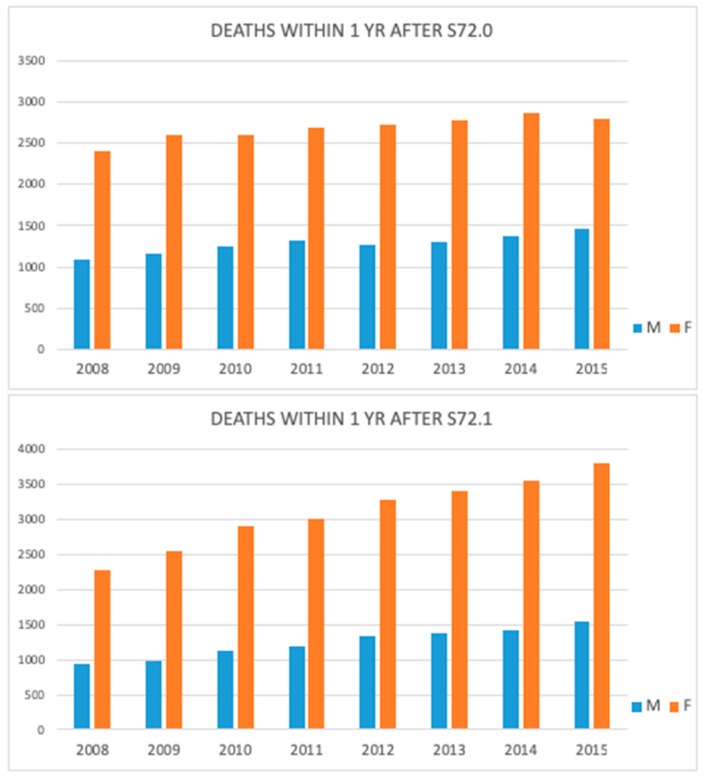
Number of deaths within one year after femoral neck (S72.0) and pertrochanteric fracture (S72.1) during the study period.

There was a median of 2911 deaths/year among men and 7193 deaths/year among women within one year after HF during the study period. Mortality increased significantly in both sexes, in both femoral neck and pertrochanteric fractures subgroups (Figure 9). Regardless of gender, there were more recorded deaths within one year after pertrochanteric fracture. 

Over the study period, mortality percentage within one year after femoral neck fracture ranged between 30.45% and 32.8% in men and 26.2%–28.04% in women. Correspondingly, it reached 30.4%–33.9% in men and 29.8%–32.06% in women for pertrochanteric fracture. 

Women aged 80–89 years were the subpopulation of the highest mortality within one year after HF; 50.7%–55.4% died after femoral neck fracture and 53%–58.5% after pertrochanteric fracture. The comparable European Union (EU) data for the same subpopulation are not available in the literature. Mortality rate, to a variable degree, was higher for men than women in the age group 50–69, regardless of fracture location.

**Figure 10:**
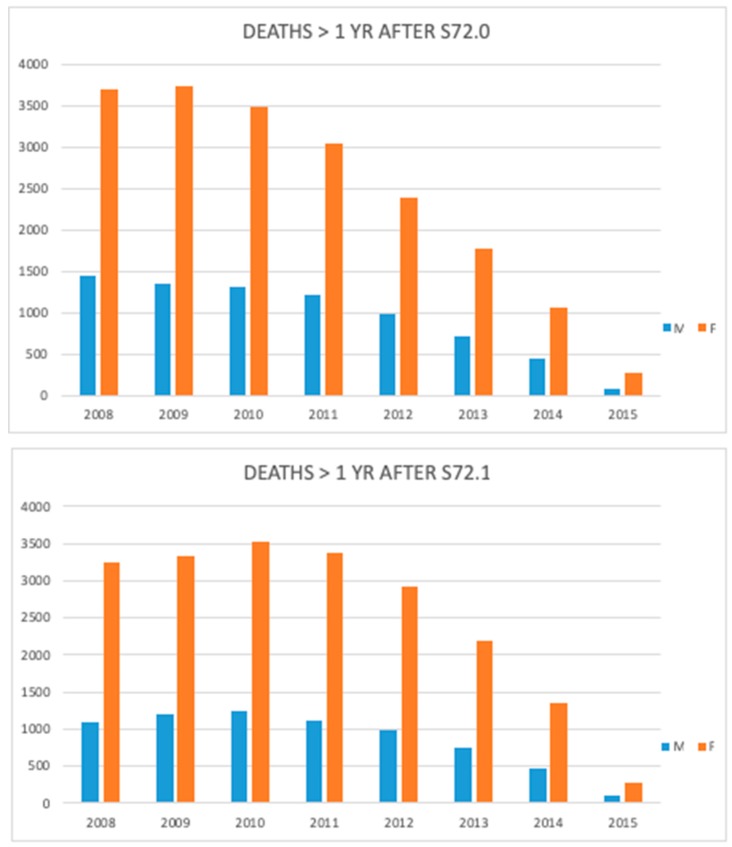
Number of deaths after one year following femoral neck (S72.0) and pertrochanteric fracture (S72.1) during the study period.

Available medical records between 2008 and 2015 showed a decreasing mortality rate in those who survived the first year after sustaining the HF (Figure 10). Trend-wise, the relationships between different age groups and between men and women remained analogous.

**Figure 11:**
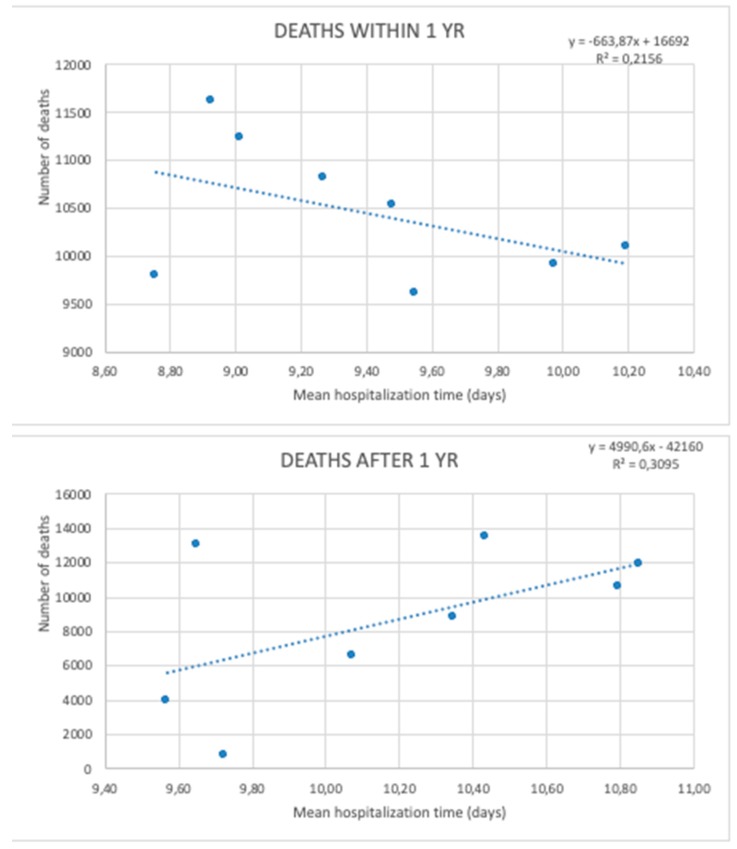
Regression models comparing hospitalization time and the number of deaths within and after one year following HF (hip fracture) during the study period.

Regression models comparing hospitalization time and the number of deaths within and after one year after HF are presented in Figure 11 (*p* < 0.05).

## 4. Discussion

**Figure 12:**
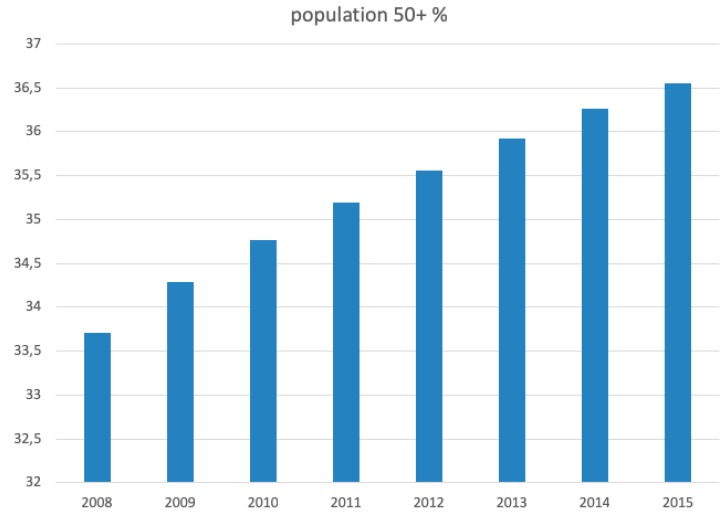
Percentage of population aged 50 years or older in Poland during the study period.

The nationwide population aged 50 years or over increased in Poland between 2008 and 2015 by over 8.5% and it accounted for more than 36% of the population in 2015, most recently reaching 37% [12] (based on data from 31 December 2018)—compared to 29% in the standard European population (Figure 12). 

### 4.1. Incidence

No published national data span more than a year in Poland [5]. Most reports come from a minority of hospitals or regional data extrapolated nationally [4,13]. Czerwinski et al. [5] published data for 2005 based on discharge codes reported to the NHF with an estimated annual number of HFs of 17,199. Based on these data, Poland was among the countries with the lowest HF rate in Europe with an overall crude incidence ratio of 89/100,000 for men and 156/100,000 in women over the age of 50. Prior reported estimations were from 109 to 283/100,000 [14,15,16].

Jaworski and Lorenc [17] estimated the annual number of HFs in Poland based on NHF data for only Mazovian Voivodeship to be 27,434 in 2007. Our data support the estimation. The present study has shown a steady increase in incidence over the study period—32,636 cases/year in 2008 to 39,783 cases/year in 2015 (a rise of 18%). 

The most recent work published by Wilk et al. [4] covers 13-year data from a single region in Southern Poland. Their observations for 2014 estimated the crude incidence rate to be 216.2 (men, 140.9; women, 276.5). We found a higher incidence rate in 2014, i.e., 445. These observations reach a value that corresponds to projections made by Wilk et al. for 2040.

Multiple reports showed substantial regional differences, even within the same country. The differences are represented not only in the HF incidence ratio, which could be the reason behind incongruences of previous estimates for Poland but also in length of stay or mortality following the fracture. The extent of these differences is further modified by gender-specific discrepancies. Our data showed that the overall female to male proximal femur fracture incidence ratio was 2.46. Previously reported national data demonstrated 1.41, while European data span from 1 to 3.36 [18,19].

Standardized rates still place Poland in the “low incidence” category [6]. Crude rates correspond closely to those of Italy [20], where the number of hip fragility fractures in 61 million Italians was 91,494. 

Rapp et al. [21] recently reported that in Europe and in most high-income countries, a decrease of HF age-standardized rates followed its rise after its peak in the 1980s and 1990s except for in the case of Germany. The increasing trend of HF incidence over the 10-year study period was reported by Mann et al. [22]. Authors comparing HF incidences and its trend using pooled data found a 30% higher rate in Austria compared to Germany. Regional hospitals frequently describe the local geographic situation in various countries [4,10,11] that does not apply to the whole country. The Harstad Injury Prevention Study [11], based on the described HFs in Northern Norway from 1994 to 2008 was compared to data from Oslo. The authors noted an exponential rise of the annual incidence of HFs from 5.8 to 349.2 per 10,000 in men and from 8.7 to 582.2 per 10,000 in women from the age group 50–54 to 90+ years. They found lower age-adjusted incidence rates of HFs in Oslo (101.0 and 37.4 in women and men, respectively). The urbanization process may also influence the incidence rate of HFs [10]. 

### 4.2. Hospitalization

There are multiple speculated causes for significant differences in lengths of hospital stay after proximal femur fracture across the countries. Most likely these are accounted for by different surgical methods, time to surgery, and rehabilitation care for different fracture types [23]. 

The EUROHOPE study (European healthcare performance study), focusing on HF, showed marked differences in hospitalization time at the national level as well as at the regional level [24]. The study gathered data from different regions of Finland, Hungary, Italy, the Netherlands, and Norway in 2007. Patients stayed at the hospital the longest in Italy (18.9 ± 1.7 days), while they were discharged after 9.8 days (±1.4 days) in Norway. In Finland and Hungary, the average length of stay was 10.4–10.9 days, which is similar to Polish data at the time. 

In Poland, patients gradually spent less time at the hospital following the fracture, reaching 9.3–9.7 days in 2015. This trend is seen across Europe and the United States [25].

There are ongoing discussions about the challenges of adequately reporting hospitalization. Hospital stays include both immediate care, postoperative stay, and rehabilitation, which reflects the period from admission to hospital to discharge to the place of residence. The ability to capture all elements is limited in Polish conditions, which is an additional challenge in comparing hospitalization time between countries and populations. The observed shortening of hospitalization can only reflect emergency care, as the report is unaware of the postoperative transfer to another hospital and the duration of rehabilitation. The differences can be significant. Access to further rehabilitation services varies considerably depending on the hospital in Poland. The pressure to transfer a large proportion of patients to postoperative/rehabilitation care elsewhere is enormous but inefficient. Access to local rehabilitation centers is very limited; sustainable home rehabilitation programs are negligible. Effective early rehabilitation and early discharge programs, supported by the local outpatient rehabilitation facility, can effectively reduce the length of stay. 

The authors have no direct source data that could explain the reasons for the reduction of hospitalization time in subsequent years in the period from 2008 to 2015. The medical care organizational changes could have contributed to shortening hospital stays. The authors hypothesize that recent changes in employment conditions of many orthopedic surgeons, i.e., shift from permanent hire to being independent contractors, accompanied by reimbursement of over-limited services by NHF could have an influence on reducing the length of stay effectively. The careful analysis of key performance indicators of this issue exceeds the scope of this work and would need a different analysis. The material available to the authors does not allow conclusions to be drawn in this regard. The length of hospitalization or length of stay may vary depending on how the data is collected. 

Hip fracture databases usually use data collected as an audit from designated hospitals. Specific questions are prepared to be answered by hospitals. There is a significant difference in the method of collecting data in the national hip fracture database (NHFD) [26,27,28] and collecting data in this study.

NHFD reports provide information that very few health systems across the world can match. The reports provide an analysis of key performance indicators. Audit databases use collected information about patients who have femoral fractures every year and combine them with information on the quality of care and results for each patient [26,27,28,29]. 

Registrations in the HF database report performance measures to compare and create consistent, high-quality care in the country and local improvements in different hospitals. Elements of the dataset are based on the recommendations contained in national and international guidelines and elements used in other international audits regarding hip fractures with quality indicators [26,28,29,30,31]. Definitions, numerators, and denominators are clearly determined in the audit.

Worldwide known hip fracture databases reports provide a detailed picture of trends in care, particularly in respect of changes in surgical and anesthetic techniques in many countries [26,27,30,32].

There are some peer-reviewed papers describing hip fracture audits and registers [26,27,28,30,31,33] form other countries (New Zealand, Australia, the Netherlands, Denmark, Ireland, Norway, United Kingdom, and others). Rikshöft [26], the Swedish National Registry of hip fracture patient care is mentioned in the literature as the first nationwide information collection. The National Hip Fracture Database (NHFD) in United Kingdom [28] is an extensive national clinical audit developed from 2007 as a result of a collaboration between the British Orthopaedic Association and the British Geriatrics Society. It is managed by the Royal College of Physicians.

The hip fracture management is well described in clinical guidelines [34], that are developed and widely accepted by specialists. There are no Polish recommendations available, which should address several issues of the HF care, namely imaging options, timing of surgery, analgesia, anesthesia, planning the theater team, surgical procedures, mobilization strategies, multidisciplinary management, and patient and carer information. The emergency department stays, the time from admission to surgery, the care and outcomes, and other information usually collected in the registers could also improve our study if available. Surgery and anesthesia data, specific fracture type, type of anesthesia, and fracture treatment method were not directly available for analysis in this study.

### 4.3. Mortality

Hip fracture is a critical and debilitating condition in older people, particularly in women. Chronic pain, reduced mobility, disability, an increasing degree of dependence, and a higher risk of death are consequences of HF.

There are systematic differences in the treatment of HFs within Europe represented by contrasting data on the relationship between the length of hospital stay and early mortality associated with HF. A longitudinal Swedish study by Nordstróm et al. [35] showed that shorter length of stay in the hospital after HF is associated with increased risk of death after hospital discharge, but only among patients with a hospital stay of 10 days or less. This association remained robust over consecutive years. In the analysis, patients that died during the hospital stay were excluded. Nikkel et al. [36] found hospitalization of 11–14 days resulted in 32% increased odds of death 30 days after discharge, compared with a maximum stay of five days. A Serbian study [37] on a consecutive series of cases found no effect of length of stay on one-year mortality, while in Northern Ireland Heyes et al. [38] found a more extended hospital stay resulted in increased readmission rate after HF.

Increased risk of death has been estimated to span beyond two years after the fracture, while approximately 25% of deaths associated with HF could be attributed to the injury itself [39].

Research showed that the highest excess mortality occurs in the first three months after the fracture and disappears by about nine months. Nevertheless, most reports focus on up to 12-month mortality. In our analysis, one-year mortality increased with age and was higher for patients with pertrochanteric fracture as compared to femoral neck fracture. Men were more likely to die within a year than women when considering the same fracture subtype. These observations are in accord with previous reports. The effect is seen as soon as within 30 days of discharge from the hospital, for reasons unknown. However, there are assumptions of a higher prevalence of chronic comorbidities preceding the fracture in men. 

The mortality rate after incident HFs in the Swedish population aged 50 years and older were 4.6-fold higher in men and 2.8-fold higher in women compared to the general population [40].

**Figure 13:**
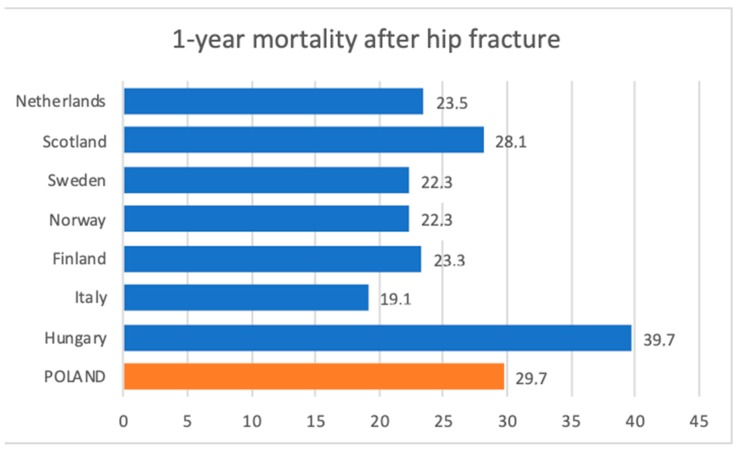
1-year mortality percentage after hip fracture—comparison between selected European countries. Calculations for Poland were made based on our data.

It was found that mortality in the first year after HF was as high as 20%–24% [41,42]. A survey conducted by Keene et al. [43] in the 1990s showed that up to 20% of patients die in the first year after HFs. We found somewhat higher mortality in Polish population with the overall percentage ranging between 30.45% and 32.8% in men and 26.2%–28.04% in women after femoral neck fracture. Correspondingly, mortality reached 30.4%–33.9% in men and 29.8%–32.06% in women for pertrochanteric fracture. Recent data, with a comparison to Poland, is presented in Figure 13. Authors argued that variations in mortality depend on country-level factors such as funding type, socioeconomic status, and availability of guidelines [18]. Elaborating on all nuances regarding different healthcare systems across Europe is beyond the scope of this paper. Several factors for discrepancies could be clearly seen. These correspond closely to areas in need of substantial improvement and are covered in this discussion.

Smith et al. [44] identified four key characteristics associated with the risk of mortality up to 12 months after HF surgery, namely abnormal ECG, cognitive impairment, age >85 years, and pre-fracture mobility (RR: 0.13; 95% CI: 0.05–0.34). A male gender, being a resident in a care institution, intra-capsular fracture type, high ASA (American Society of Anesthesiologists) grade, and high Charlson comorbidity score on admission were statistically significant pre-fracture predictors of increased mortality as well. 

Söderqvist et al. [45] determined preoperative factors associated with mortality, evaluated the combined use of the American Society of Anesthesiologists (ASA) and the Short Portable Mental Status Questionnaire (SPMSQ) to identify patients with an increased mortality rate, and created a predictive model to assess the mortality risk after HF surgery. They observed that the mortality rate of 1944 consecutive patients during the acute hospitalization period was 4%, at four months after discharge 16%, and at 24 months 38%. The critical mortality risk factors were high ASA scores, low SPMSQ scores, high age, and male gender. Pioli et al. [46] investigated the one-year mortality risk associated with HF in older adults. The effects of comorbidity and functional impairment on long-term mortality after HF led to a cumulative mortality of 24% at 12 months. 

Long-term excess mortality in patients with HF could be driven by differences in health status that existed before HF. Patients with HF are, on average, more functionally impaired and have more comorbid conditions than similar-aged patients without HF. Only less than half of the HF survivors regain their previous level of function [43]. Disability and nursing home use due to the loss of function and independence among survivors is significant. Forty percent of patients are unable to walk independently, and 60% require assistance a year later [47]. Because of these losses, 33% are entirely dependent or in a nursing home in the year following a HF [42,48]. 

Prospective studies of functional outcomes after fracture indicate that older adults develop new functional impairments and loss in quality of life that frequently persist at one year [49,50,51].

Despite advancements in expertise and medical technology, there are areas in need of considerable improvement at all systemic levels. The overall increase is funding does not need any further comments. Policymakers should incentivize quality and long-term outcomes, to ensure that rehabilitation and community-based care are included in a patient’s pathway. Surgery should be performed on the day of, or the day after, admission, preferably in the regular working hours. Effective rehabilitation with early discharge, followed by sustainable out-patient care should follow. Primary and secondary fracture prevention focused on reduction in the incidence of falls and antiresorptive medication should not only improve the care of hip fracture but also diminish its incidence. We should develop a culture of continuous improvement, and by accurate collection of data, evaluate our performance. Finally, the development of national guidelines for HF management and prevention would ensue. 

The strength of the study was to use the database based on mandatory reports from all hospitals around the country. There is a gap between the items of mandatory data reported and the data collected in specialized registers. National hip fracture audits or databases are developed in several countries. So far, no audit-like registry is available in Poland, unlike other countries. The audit database analysis could show the differences between hospitals in HF orthopedic management. The guidelines [34] state that every patient over 70 years should receive orthogeriatric management during admission. Orthogeriatric management is not well recognized in Poland [52]. 

Our study has some limitations. The analysis regards only patients aged 50 years or older who were discharged from the hospital with ICD10 code of S72.0 and S72.1. Some of the patients at this age, with various proportions, might have suffered from high impact energy trauma resulting in proximal femur fracture not related to osteoporosis. Patients younger than 50 years might have had osteoporosis and sustained a common disease-related fracture. The nature of these limitations is coding-related and could only be overcome with thorough and reliable coding practices.

Nevertheless, the study has some strengths. It is the first study based on national data spanning eight years. It evaluates medium and long-term mortality after proximal femur fracture and hospitalization time in the same period, which are indirect indices of the quality of provided care.

The hip fracture epidemiology and mortality were the aim of this study instead of hip fracture care in Poland due to the lack of the audit database. A limitation of the HF audits concerns the data obtained from designated hospitals. In our study, data from all hospitals in the country reimbursed by the National Health Fund were analyzed. The revealed limitations of the hip fracture audits show the case ascertainment from 58% up to 100%. The study presented here is based on the completeness of the collected data from all documented hip fractures treated in the National Healthcare Hospitals. The extent of these data reflect those necessary for the treatment reimbursement from the NHF. 

The authors believe that research can be an essential premise to initiate work on creating a registry and join the international network. The HF database could be supervised by a multidisciplinary commission in which medical associations involved in the treatment of proximal femoral fractures in Poland will be represented. The scientific committee should decide on the content of the HF database and be responsible for developing methodologically sound quality indicators.

In conclusion hip fractures in Poland are increasingly more prevalent in the aging population. The disadvantageous trend observed in this study accounting for relatively higher one-year mortality after hip fracture when compared to other European countries should be considered a significant public health and economic concern. Hip fracture is related to the development of other harmful complications, such as disability, depression, cardiovascular diseases, and finally higher mortality rate. Improvement of the hospital and out-patient care would require an implementation of a national fracture prevention program.

## Figures and Tables

**Table 1:** The incidence rate of the femoral neck and pertrochanteric fractures per 100,000 population by year. FRX—fracture.

	Year	Total Incidence/100,000	Incidence in Men/100,000	Incidence in Women/100,000
Femoral Neck FRX	2008	119	67	167
	2009	146	75	212
	2010	113	62	161
	2011	124	68	176
	2012	147	78	211
	2013	205	108	295
	2014	220	117	317
	2015	207	125	283
Pertrochanteric FRX	2008	73	42	102
	2009	104	53	152
	2010	98	52	141
	2011	115	63	163
	2012	143	82	201
	2013	212	108	309
	2014	225	114	329
	2015	237	121	346

**Table 2:** Polish incidence rate of proximal femur (“hip”) fracture per 100,000 population by the year, standardized to the European population.

Proximal Femur Fracture	Total Incidence/100,000	Incidence in Men/100,000	Incidence in Women/100,000
2008	56	32	78
2009	73	37	106
2010	61	33	88
2011	69	38	98
2012	84	46	119
2013	121	63	175
2014	129	67	187
2015	129	71	183
